# *TMPRSS2-* Driven *ERG* Expression *In Vivo* Increases Self-Renewal and Maintains Expression in a Castration Resistant Subpopulation

**DOI:** 10.1371/journal.pone.0041668

**Published:** 2012-07-30

**Authors:** Orla M. Casey, Lei Fang, Paul G. Hynes, Wassim G. Abou-Kheir, Philip L. Martin, Heather S. Tillman, Gyorgy Petrovics, Hibah O. Awwad, Yvona Ward, Ross Lake, Luhua Zhang, Kathleen Kelly

**Affiliations:** 1 Cell and Cancer Biology Branch, Center for Cancer Research, National Cancer Institute, National Institutes of Health, Bethesda, Maryland, United States of America; 2 Department of Surgery, Center for Prostate Disease Research, Uniformed Services University of the Health Sciences, Rockville, Maryland, United States of America; Northwestern University, United States of States

## Abstract

Genomic rearrangements commonly occur in many types of cancers and often initiate or alter the progression of disease. Here we describe an in vivo mouse model that recapitulates the most frequent rearrangement in prostate cancer, the fusion of the promoter region of *TMPRSS2* with the coding region of the transcription factor, *ERG*. A recombinant bacterial artificial chromosome including an extended *TMPRSS2* promoter driving genomic *ERG* was constructed and used for transgenesis in mice. *TMPRSS2-ERG* expression was evaluated in tissue sections and FACS-fractionated prostate cell populations. In addition to the anticipated expression in luminal cells, *TMPRSS2-ERG* was similarly expressed in the Sca-1^hi^/EpCAM^+^ basal/progenitor fraction, where expanded numbers of clonogenic self-renewing progenitors were found, as assayed by in vitro sphere formation. These clonogenic cells increased intrinsic self renewal in subsequent generations. In addition, ERG dependent self-renewal and invasion in vitro was demonstrated in prostate cell lines derived from the model. Clinical studies have suggested that the *TMPRSS2-ERG* translocation occurs early in prostate cancer development. In the model described here, the presence of the *TMPRSS2-ERG* fusion alone was not transforming but synergized with heterozygous *Pten* deletion to promote PIN. Taken together, these data suggest that one function of *TMPRSS2-ERG* is the expansion of self-renewing cells, which may serve as targets for subsequent mutations. Primary prostate epithelial cells demonstrated increased post transcriptional turnover of ERG compared to the TMPRSS2-ERG positive VCaP cell line, originally isolated from a prostate cancer metastasis. Finally, we determined that *TMPRSS2-ERG* expression occurred in both castration-sensitive and resistant prostate epithelial subpopulations, suggesting the existence of androgen-independent mechanisms of TMPRSS2 expression in prostate epithelium.

## Introduction

Prostate adenocarcinoma is believed to develop from early precursor lesions known as prostatic intraepithelial neoplasia (PIN) [1]. A majority of prostate cancers has a pronounced luminal phenotype and are classified histologically as acinar adenocarcinomas. In addition to the major luminal phenotype, there exists heterogeneity in the form of minor populations of tumor cells as revealed by in situ staining and by fractionation of live tumor cell suspensions [2], [3], [4], [5]. The role of various populations in contributing to the development of tumors and/or their subsequent progression to metastatic or castration resistant cancers is an area of intense interest. Minor subpopulations observed within human and mouse prostate cancers have been shown to demonstrate correlated properties of self-renewal, production of differentiated progeny, and growth as transformed lesions upon transplantation [6], [7].

Chromosomal translocations that create cell-type specific fusion genes with oncogenic activity occur in various types of cancers [8]. The most frequent genomic rearrangement in prostate cancer is fusion of the Ets transcription factor, Ets related gene (ERG), with the promoter of the highly-expressed transmembrane protease serine 2 (TMPRSS2) gene. Approximately 50% of prostate cancer samples from PSA screened cohorts contain a TMPRSS2-ERG fusion gene [9]. An extensive evaluation of whole mount prostates has shown a nearly 100% concordance of ERG positive PIN with ERG positive carcinoma [10]. The lower concordance of ERG positive carcinoma and PIN in tissue microarrays may be in part the consequence of multi-focal tumor heterogeneity [11]. In addition, it appears that TMPRSS2-ERG fusion also can be an initiating or pre-malignant event as implied by the rare observations of TMPRSS2-ERG fusions in low grade lesions including atypia and low grade PIN [10], [12]. Taken together, these clinical data support the occurrence of TMPRSS2-ERG translocation as an early event in prostate cancer that is subsequently selected during malignant transformation.

The functional role of ERG overexpression is of obvious interest. As one approach to investigating this question, several mouse models have been analyzed in which either full-length or N-terminal truncations of ERG cDNA’s were expressed from a modified probasin ARR_2_-*probasin* (PB) promoter. Conflicting results have been reported from such studies. Two studies described epithelial hyperplasia and focal PIN lesions [13], [14] while two others found no significant pathological changes [15], [16]. The latter studies, however, found accelerated transformation resulting from ERG over-expression in combination with heterozygous *Pten* deletions. Another approach has used lentivirus transduction of ubiquitin C promoter driven ERG cDNA into suspensions of primary mouse prostate epithelial cells, followed by transplantation in combination with embryonic urogenital mesenchyme under the kidney capsule [17]. Such transplanted cells developed into glands with focal PIN lesions. Thus, both the infection-transplantation and transgenic mouse models are consistent with clinical data suggesting that ERG plays a role in early events leading to prostate neoplasia.

ERG also appears to contribute to invasion, which is particularly evident in cell lines expressing *ERG* ectopically [14], [15], [18]. In transduced primary prostate epithelial cells reimplanted in vivo, ectopic ERG in combination with either activated AKT or androgen receptor, but not ERG alone, produced lesions with invasive features [17]. Also, transgenic ERG expression combined with heterozygous *Pten* deletion led to adenocarcinoma development in one study [15] but not another [16].

Taken together, the above range of results suggests that ERG function is potentially affected by various factors. It seems likely that ERG function will be influenced by expression level. In addition, ERG fusions are formed by rearrangements that result in variable inclusion of ERG N-terminal sequences, and the ERG gene body is subject to alternative splicing, leading to various isoforms 9,19,20. Some isoforms appear to encode different relative levels of functional activity [20]. Finally, we expect that cellular context will be important for observing certain ERG functions. This is especially true in vivo where minor populations, that may be distinct from differentiated prostate luminal tumor cells, probably play a role in tumor development [7]. The lineage specificity of the *TMPRSS2* promoter is a major factor determining context-dependent ERG expression from the fusion gene. The *TMPRSS2* promoter has been investigated in various prostate cancer cell lines, where it has been shown to be highly expressed in luminal cells and positively regulated by androgen receptor [21], [22]. However, relatively little is known about the lineage specificity and androgen regulation of the *TMPRSS2* promoter in vivo.

Genetic events that initiate or contribute to early transformation likely target self –renewing cells, in which subsequent genetic and epigenetic abnormalities can accumulate. ERG functions in hematopoietic stem cells (HSC) as one of a small number of transcription factors responsible for stem cell maintenance, the regulation of balanced self-renewal and committed progenitor production [23], [24]. When over-expression of ERG in prostate epithelial cells resulted in focal PIN lesions, there also was evidence of abnormal lineage differentiation [13], [17]. Thus, we hypothesized that one effect of TMPRSS2-ERG expression in preneoplastic cells is to modify clonogenic self renewal. To develop a mouse model that recapitulates many features of the translocation in human prostatic tissue, we used recombineering to produce a bacterial artificial chromosome (BAC) harboring 25 kb of the human *TMPRSS2* promoter plus *TMPRSS2* exons 1 and 2 juxtaposed to the genomic region downstream of a common breakpoint region of human *ERG* [25]. The recombinant BAC construct was subsequently used to produce transgenic mouse strains. Expression from the legitimate *TMPRSS2* promoter provides the potential to observe faithful cell expression profiles and physiological regulation. Furthermore, the use of the genomic *ERG* locus maintains potential splicing and microRNA-dependent regulatory mechanisms. Finally, single copy transgenes are usually incorporated during BAC transgenesis, minimizing unnatural overexpression.

This study aims to examine the expression patterns and function of TMPRSS2-ERG in normal prostate and in early neoplastic prostate lesions. *TMPRSS2-ERG* was found to be expressed in basal/progenitor as well as luminal cells, and *TMPRSS2-*driven *ERG* expression in transgenic prostates resulted in increased clonogenic sphere forming activity. *TMPRSS2-*driven *ERG* expression in primary prostate epithelium was found to be partially castration-resistant, implying the potential for androgen-independent *TMPRSS2* promoter activity in prostate epithelial subpopulations.

## Results

### The Genomic TMPRSS2-ERG BAC Model Produces Accurately Spliced Transcripts and Expressed Proteins

**Figure 1:**
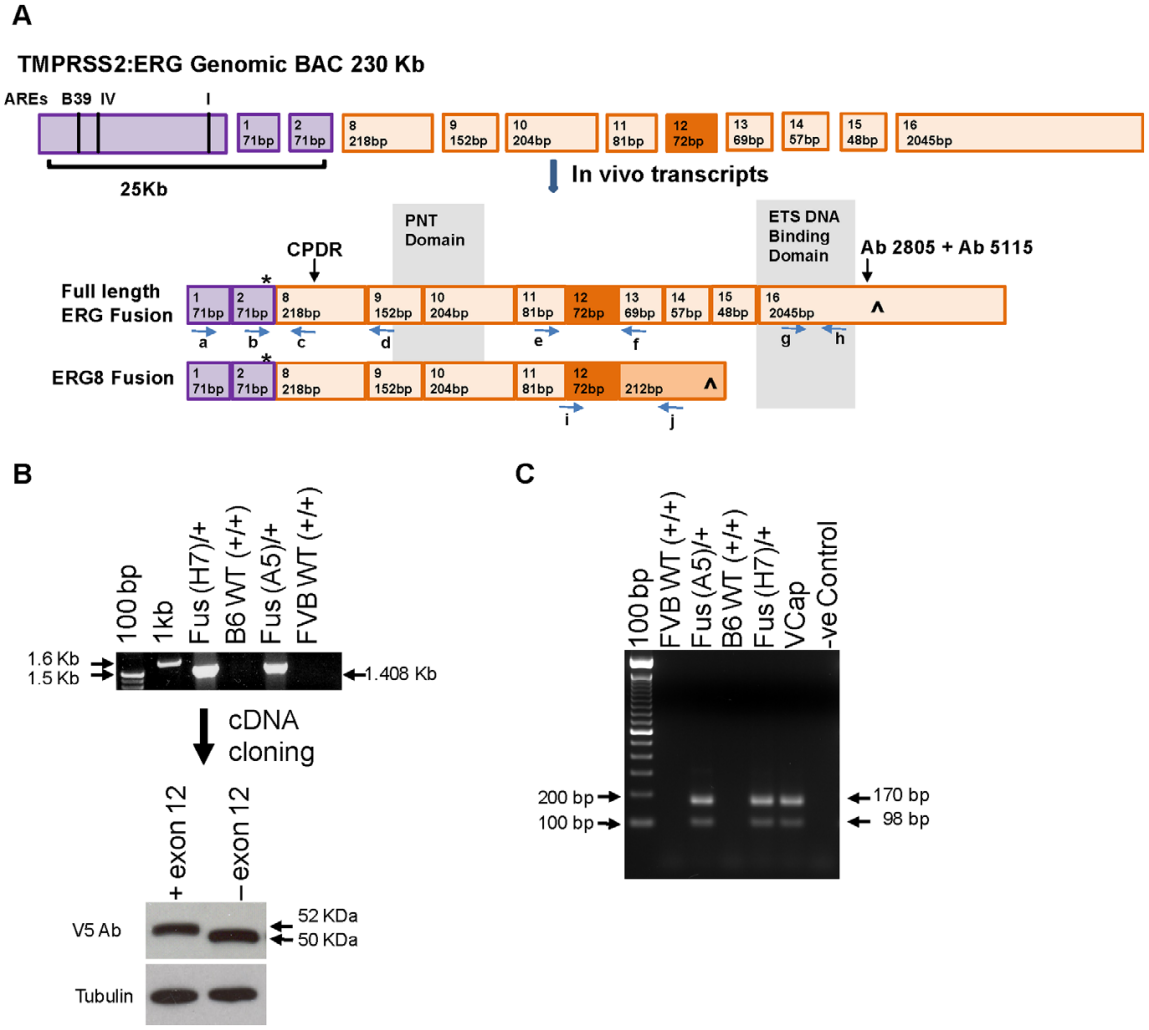
Characterization of a BAC TMPRSS2-ERG fusion model displaying clinically relevant alternatively spliced transcripts. Fusion (Fus) A5 and Fus H7 refer to independently generated BAC transgenic lines. (A) Schematic representation of the recombined human TMPRSS2-ERG genomic BAC and the resulting characterized transcripts. Exons are numbered 1–16 and the functional domains are indicated, * denotes potential start and ∧ stop codons. PCR primers are labeled a–j. (B) Top panel: RT-PCR example using primers a/h showing the absence of mRNA transcripts in wild type (WT) and presence in the transgenic lines. Subsequent cDNA cloning and sequencing revealed two variants that differ by the presence of exon 12. Lower panel: Western blot detection of individual V5 tagged proteins following transient transfection of Cos 7 cells with the indicated fusion ERG cDNA inserts in expression vectors. (C) RT-PCR with primers e/f and using total RNA isolated from organoid cultures or the VCaP cell line.

Recombineering was used to construct a bacterial artificial chromosome (BAC) that incorporated a 25 Kb human *TMPRSS2* promoter plus exons 1 and 2 adjacent to the human *ERG* genomic region downstream of intron 7/exon 8 (Figure 1A); the exon nomenclature used here is from Owczarek *et al.,* [26]. The *TMPRSS2* upstream region contained previously-mapped AR binding sites [22]. The BAC construct mimics a relatively common class of TMPRSS2-ERG fusion (type VI) found in clinical samples [9], which has been associated with a more aggressive phenotype, including increased seminal vesicle invasion and early PSA recurrence following treatment [27]. An ATG start codon in exon 2 of *TMPRSS2* is in frame with *ERG* exon 8, leading to the formation of a fusion protein. Transgenic animals were generated in the FVB and C57/BL6 backgrounds, and one line from each background, A5 and H7, respectively, was selected for further investigation. cDNA clones of fusion transcripts derived from transgenic prostates demonstrated the expected sequences and the existence of accurately-spliced transcripts with and without *ERG* exon 12 (Figure 1B). RT-PCR analysis with primers spanning exon 12 showed similar alternative splicing patterns for transgenic prostates and the VCaP cell line (Figure 1C), which have been shown to be comparable to clinical samples [19], [20]. Transient transfection of Cos 7 cells with cDNA constructs of the ± exon 12 variants produced single bands whose molecular weights were consistent with initiation at the *TMPRSS2* ATG (Figure 1B).

**Figure 2:**
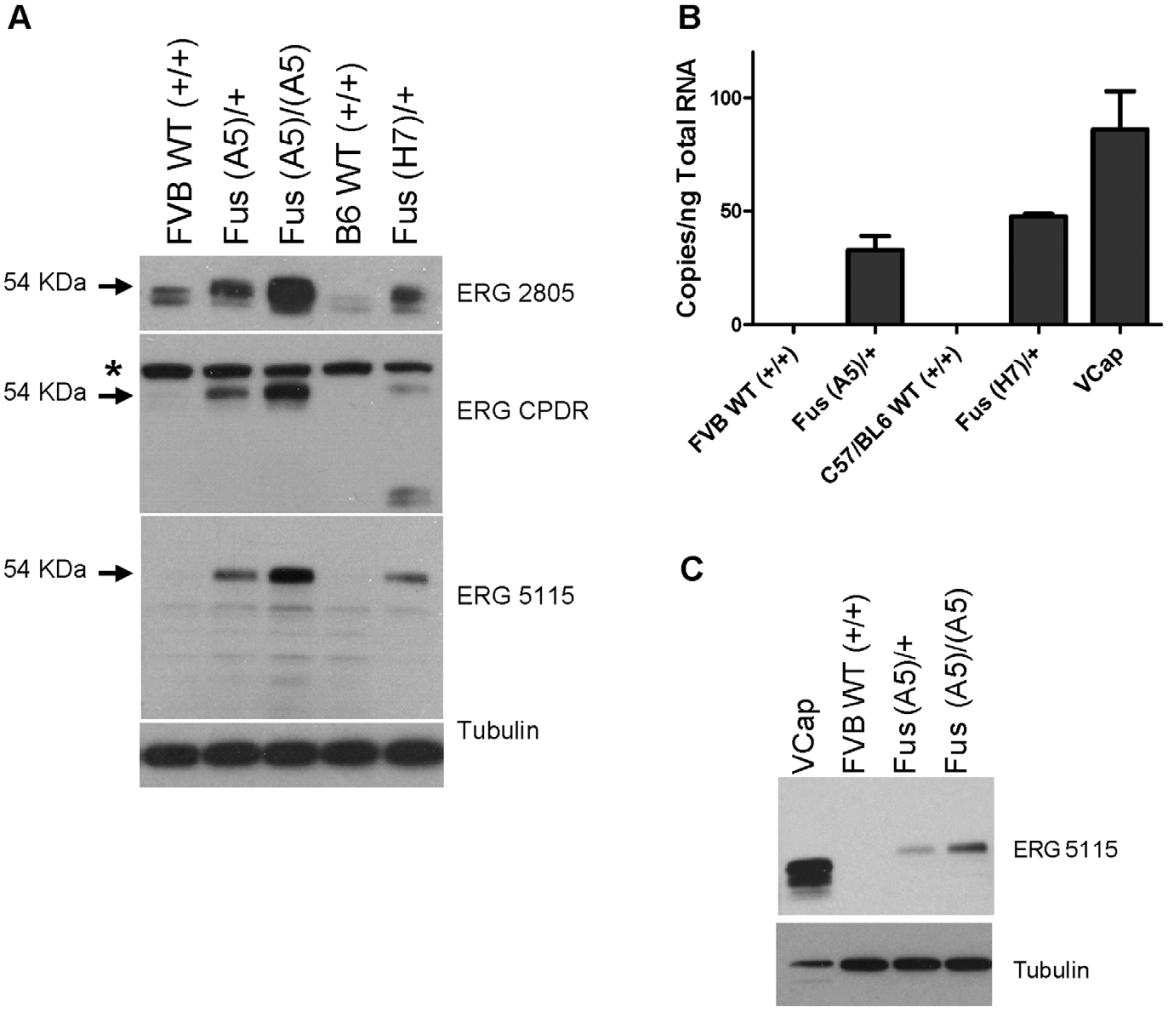
Characterization of ERG expression within the transgenic lines. (A) Western blot of ERG expression in WT and fusion prostate organoid cultures. * denotes non-specific signal. (B) Quantification of ERG expression level within organoid cultures (n = 3) using primers e/f and ERG FAM (Table S1). Comparison to VCaP (two independent replicates) is shown where copies/ng total RNA were derived using a standard curve for ERG copy number and samples were normalization to Gapdh. (C) Western blot comparing relative Erg protein expression in the VCaP cell line (2 ug of protein loaded) and organoid cultures (20 ug protein loaded per sample), using Ab ERG 5115.

Protein expression in transgenic animals was confirmed by Western blot analysis of lysates from prostate epithelial organoids (Figure 2A). Western blots are shown using rabbit monoclonal antibodies 2805 and 5115 directed to the ERG C-terminus and mouse monoclonal antibody ERG [10] directed to the ERG N-terminus. To investigate *ERG* transcript levels, quantitative reverse transcription-PCR was performed with primers that have been previously used for the analysis of VCaP cells and laser capture microdissected prostate cancer [19]. Independently-maintained VCaP cultures from two laboratories were used for comparison. As shown in Figure 2B, transgenic organoids and VCaP cells expressed approximately similar levels of *ERG*. A comparison of ERG protein levels demonstrated noticeably higher steady-state levels in the VCaP line than prostate organoids (Figure 2C), implying a post-transcriptional regulatory mechanism that is operationally different in these primary cells as compared to the VCaP adenocarcinoma cell line.

### TMPRSS2-ERG Synergizes with *Pten* but not *Nkx3.1* Loss to Promote mPIN

Fusion transgenic prostates, A5 (FVB) and H7 (C57/BL6) did not show abnormal histological features even at 15 months of age, demonstrating that increased TMPRSS2-driven ERG expression in the prostate is insufficient to initiate overt oncogenesis. To determine the functional activity of the TMPRSS2-ERG BAC with respect to oncogenesis, we performed crosses with mice harboring weakly transforming genetic lesions. As Pb-driven *ERG* transgenic models have been shown to synergize with *Pten* loss [15], [16], and loss of Nkx3.1 has been reported to co segregate with the TMPRSS2-ERG fusion [28], we investigated the phenotypes of TMPRSS2-driven ERG expression in combination with heterozygous loss of *Pten* and homozygous loss of *Nkx3.1*. A pathological evaluation at 28 weeks of fusion A5 mice containing a heterozygous deletion of *Pten* compared to *Pten^+/-^* mice demonstrated increased total numbers and severity of mPIN lesions associated with TMPRSS2-ERG (Table 1). However, this difference in mPIN formation was not maintained at 1 year and did not lead to more rapid progression to adenocarcinoma, indicating that the combination of TMPRSS2-ERG with *Pten^+/-^* contributes to the formation of early lesions but does not promote progression in an FVB/C57BL6 F1 background. The presence of heterozygous Pten allele deletion on a C57/BL6 background led to the more rapid development of mPIN and adenocarcinoma lesions as compared to an FVB background, which decreased the sensitivity of observing a synergistic effect between ERG and Pten*^+/-^*. By contrast, the combination of TMPRSS2-ERG and homozygous deletion of *Nkx3.1* did not synergize to produce increased numbers or severity in mPIN lesions (Table 1), revealing specificity for the *TMPRSS2-ERG* interaction with *Pten* loss. These data demonstrate functional activity of *TMPRSS2*-driven ERG expression in the transgenic lines analyzed here. Immunohistochemical staining with antibodies directed toward AR, KRT5, KRT8, TP63, and pAKT showed no obvious differences in mPIN lesions developing in *TMPRSS2-ERG* transgenic as compared to non-transgenic mice.

**Table 1:** Description of lesion development when TMPRSS2-ERG is crossed with *Nkx3.1^-/-^* and *Pten* ^+/-^ transgenic lines.

	Hyperplasia	PIN	Adenocarcinoma
*FVB Pten* ^+/-^(28 weeks)	7/7 (4.29)	2/7 (0.57)	0/7
A5/*Pten* ^+/-^(28 weeks)	8/8 (3.88)	7/8 (3.14^a^)	0/8
*FVB Pten* ^+/-^(52 weeks)	4/4 (17.25)	4/4 (13.5)	2/4 (5.0)
A5/*Pten* ^+/-^(52 weeks)	4/4 (21)	4/4 (18.75)	0/4 (0)
*FVB Nkx3.1^-/-^*(28 weeks)	5/5 (79.6)	3/5 (4.4^b^)	0/5
A5/*Nkx3.1^-/-^*(28 weeks)	5/5 (78)	5/5 (10^b^)	0/5

The numbers of mice displaying the indicated lesions relative to the number of mice analyzed is shown. The values in parentheses correspond to the average number of lesions per prostate where a significant increase in mPIN was observed in A5/*Pten* ^+/-^ compared to *Pten* ^+/-^ at 28 weeks.

a *p*≤0.05.

b Numbers of lesions per animal ranged from 0–17*^b^ p* = 0.15.

### TMPRSS2-ERG is Expressed in Luminal and Basal/Progenitor Cells

**Figure 3:**
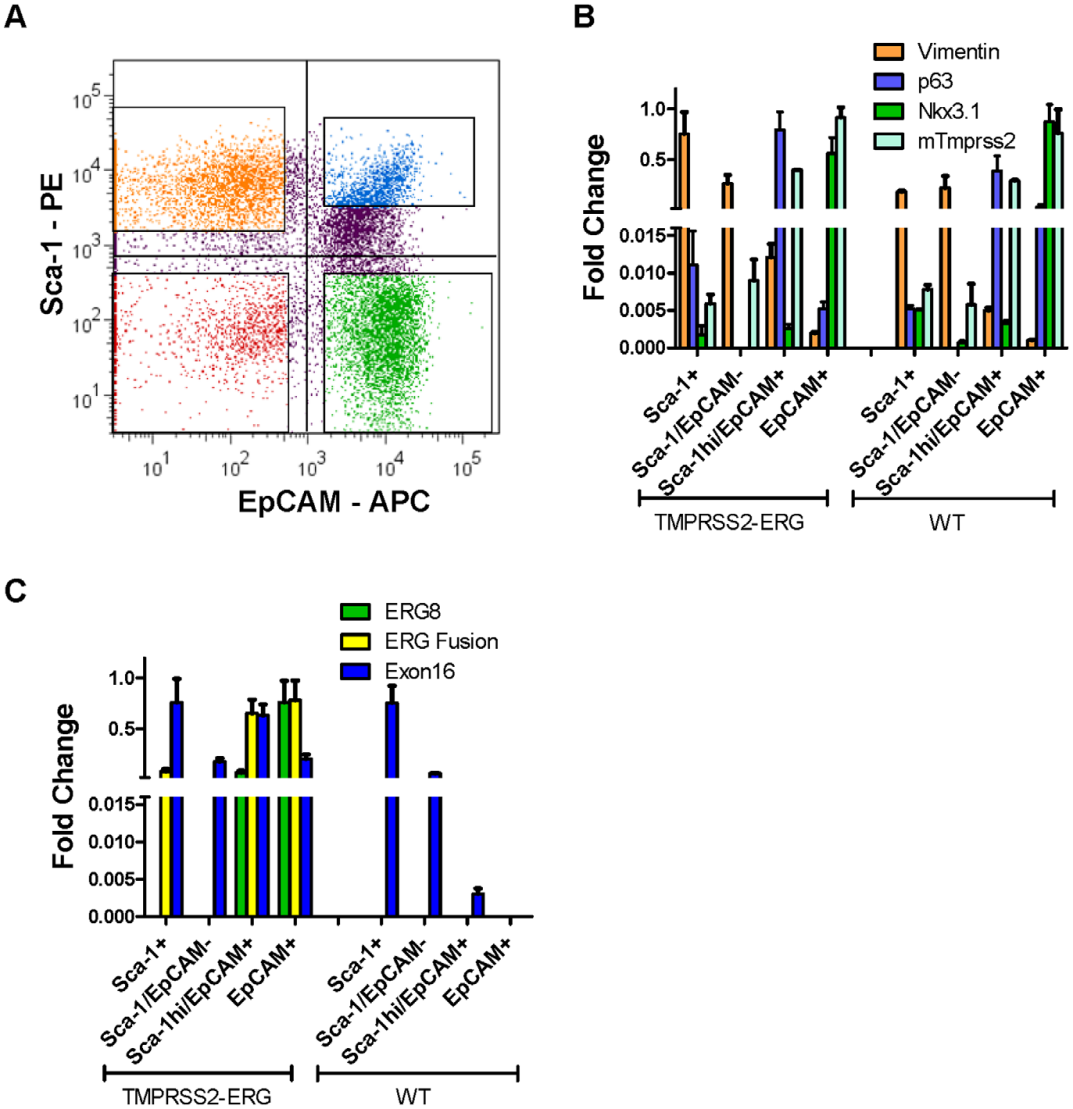
Expression of TMPRSS2-ERG in subpopulations of primary prostate epithelial cells fractionated by FACS. (A) Scatter plot of lineage negative (Lin-) prostate populations labeled for Sca-1 and EpCAM. The gated regions for the four isolated populations are shown. Upper left quadrant: Sca-1+; lower left quadrant: Sca-1/EpCAM-; upper right quadrant: Sca-1hi/EpCAM+; lower right quadrant: EpCAM+. (B) Representative examples are shown for QRT-PCR determined expression in WT and A5 RNA samples of the various fractions. Expression values were normalized to *Gapdh*, and the highest resulting expression value for each primer pair was set to 1. (C) Isoform-specific expression of ERG is shown for ERG fusion, ERG8, and ERG exon 16 using primer pairs b/d, i/j, and g/h, respectively (Figure 1A).

To establish the pattern of ERG expression in the prostate, single cell suspensions of wild type and transgenic prostates were fractionated using FACS. Lineage negative epithelial and stromal cells were fractionated using EpCAM (CD326) and Sca-1 (Figure 3A). As determined here by mRNA expression of lineage markers (Figure 3B), fractions almost exclusively contained the following populations: the EpCAM^−^\Sca-1^+/-^ fractions contained *Vim^+^* stromal cells; the EpCAM^+^\Sca-1^hi^ fraction contained *Tp63^+^* basal epithelial cells, and the EpCAM^+^\Sca-1^−^ fraction contained *Nkx3.1* ^+^ luminal epithelial cells. The Sca-1^hi^ fraction previously has been shown to contain basal cells and stem/progenitor cells capable of prostate gland regeneration [29], [30]. Of interest, mouse *Tmprss2* expression was distributed between basal and luminal epithelial cell fractions. As shown in Figure 3C, primer sets specific for the TMPRSS2-ERG fusion transcript demonstrated expression in the basal/progenitor and luminal cell fractions. In addition, we analyzed various alternative *ERG* transcripts, including ERG8, which encodes a truncated variant of ERG lacking the Ets binding domain, and *ERG* exon 16, which measures full-length *ERG* [19], [20]. In a study of clinical prostate samples, the ERG8 transcript was found to be more abundant then full length ERG [19], and consequently these variants were of particular interest. The ERG8 primers are specific for human RNA, while exon 16 primers cross-react with human and endogenous mouse *Erg*. As shown for wild type mice in Figure 3C, the majority of the nontransgenic endogenous *Erg* exon 16 was in the non-epithelial fractions. Transgenic *ERG* exon 16 was expressed in both basal and luminal epithelial cell fractions. ERG8 was expressed higher in luminal as compared to basal cells, consistent with previous observations of high relative levels of ERG8 in luminal prostate cancers [19], [20]. As described in Figure S1, RNA encoding *Fli-1,* an ERG homologue for which antibody cross-reactivity with ERG has been described [31], was mainly expressed in non-epithelial fractions.

**Figure 4:**
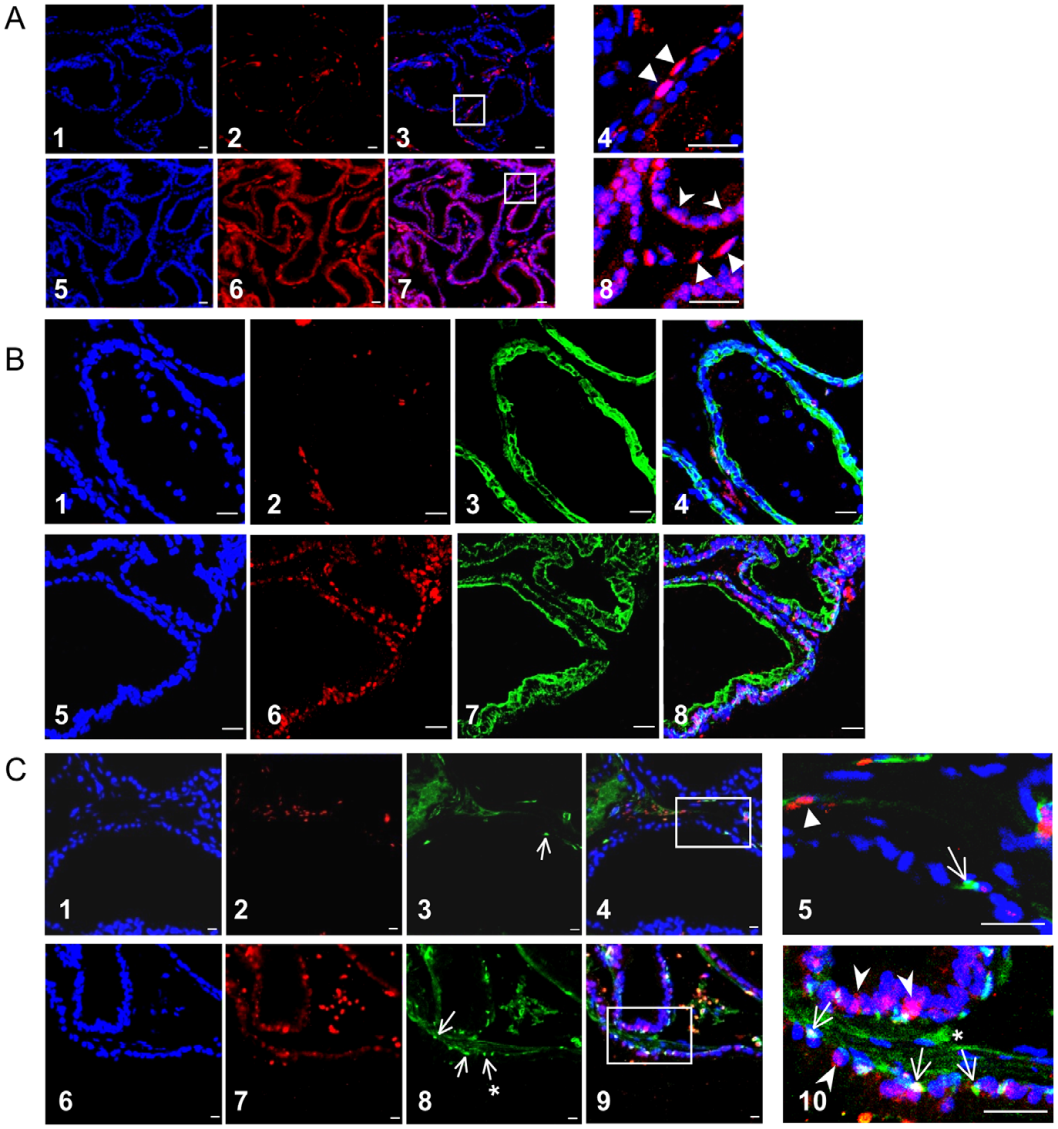
Expression of TMPRSS2 ERG in basal and luminal prostate cells. (A) Images 1–8. WT (1–4) and A5 (5–8) prostate sections stained with Dapi (1&5) and ERG Ab 2805 (2&6). Composite images (3&7) show increased ERG staining in transgenic compared to WT. Enlarged regions (4&8) show ERG positive endothelial cells found in WT and transgenic tissue, indicated by triangle symbol, and ERG positive epithelial cells, present in A5 tissue, indicated by arrowheads. (B) Images 1–8. WT (1–4) and A5 (5–8) prostate sections stained with Dapi (1&5), ERG Ab 2805 (2&6) and KRT8 (3&7). Composite images (4&8) show KRT8/ERG positive luminal cells. (C) Images 1–10. WT (1–5) and A5 (6–10) prostate sections stained with Dapi (1&6), ERG Ab 2805 (2&7) and TP63 (3&8) where arrows indicate positive staining. Composite images (4&9) and enlarged regions (5&10) show ERG positive basal cells are present in A5 prostate. Twenty three percent of TP63^+^cells co-stained for ERG (5 fields). Symbols correspond to the following cells/stains; Triangle symbol, ERG positive endothelial cell; Arrows, TP63 positive basal cells where absence of an asterix corresponds to an ERG positive cell and presence corresponds to an ERG negative basal cell; Arrowheads, ERG positive luminal cells. For all images, scale bars = 20 um.

Tissue sections from wild type and transgenic fusion mice were stained with Ab2805, as this antibody has been validated in histological analysis of human *TMPRSS2-ERG* prostate cancers [32]. Increased ERG expression was observed in TMPRSS2-ERG transgenic prostate epithelial cells compared to WT, where positive staining was observed in endothelial cells (Figure 4A). ERG was expressed in the majority of luminal cells as demonstrated by co-staining for KRT8 (Figure 4B). Interestingly, co-staining for ERG and TP63 revealed a subpopulation of TP63^+^ cells, located in a basal position and with typical basal cell morphology, that co-stained for nuclear ERG. TP63^+^/ERG^+^ cells were not observed in sections from non-transgenic animals (Figure 4C).

Taken together with the FACS fractionation data, we conclude that transgenic *TMPRSS2-ERG* is expressed in mature luminal and a fraction of basal cells. These data suggest that an extended TMPRSS2 promoter region can be expressed in multiple prostate epithelial cell types.

### Increased Progenitor Activity and a Unique Subpopulation is Observed in Fusion Versus WT Prostate Cells

**Figure 5:**
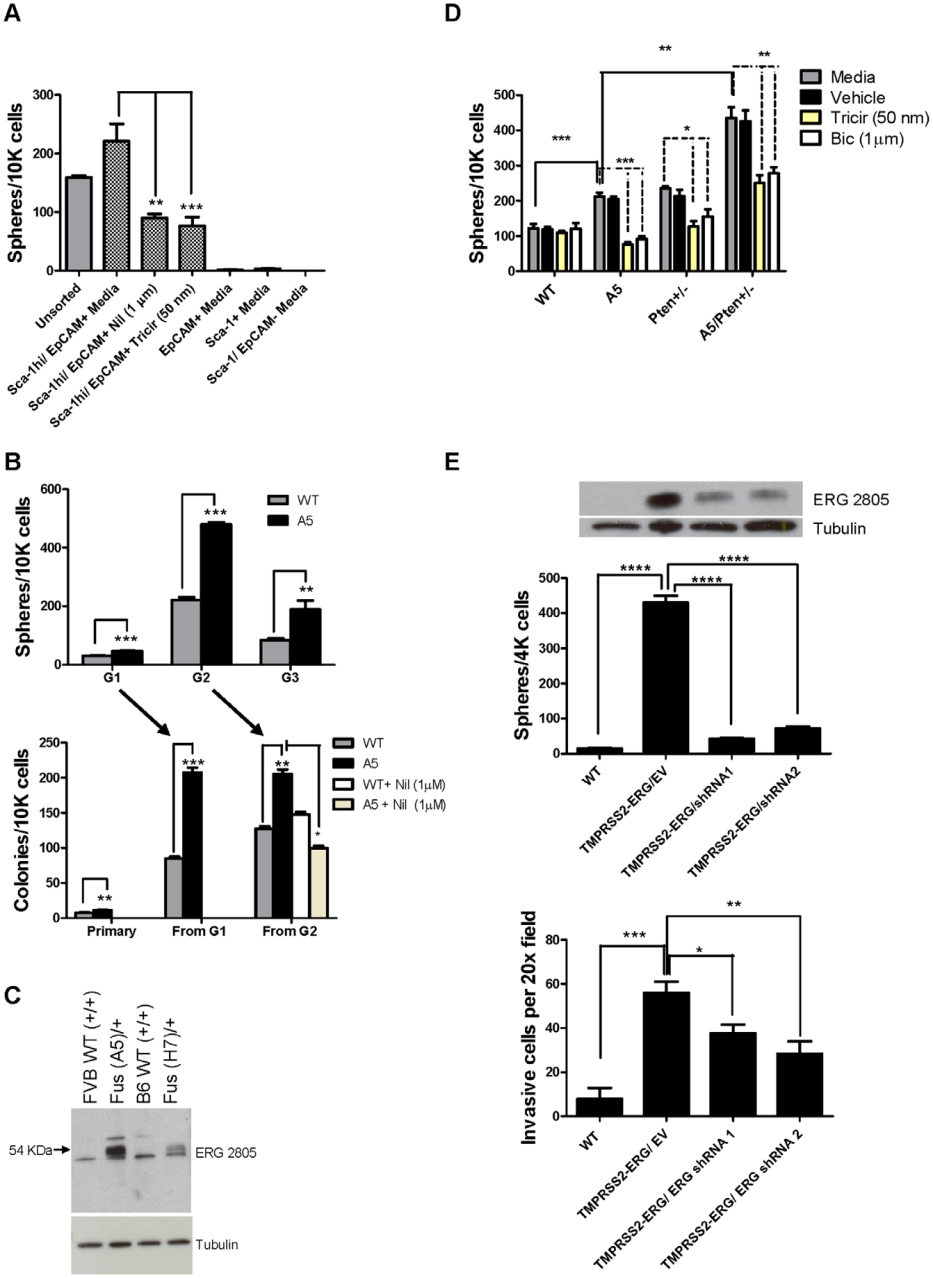
TMPRSS2-ERG expression is correlated with and determines the presence of distinct self-renewing prostate cells. (A) *TMPRSS2-ERG* spheres derived from progenitors in the Sca-1/EpCAM+ FACS fraction are sensitive to AR and AKT inhibitors. FACS sorted cells, derived from H7 transgenic prostates, were plated in triplicate in Matrigel™ for each of the four populations isolated using Sca-1/EpCAM. Shown is the Sca-1hi/EpCAM+ fraction, which contains the progenitor cells, treated with the AR antagonist Nilutamide (Nil) or the AKT inhibitor Triciribine (Tricir), at the initiation of culture. The number of spheres formed was counted at day 11. Error bars correspond to ± s.d. (B) Progenitor activity measured using colony and sphere forming assays in fusion transgenic compared to WT prostates. Propagation was performed where spheres were dissociated and serially replated to quantify progenitor activity in the next generation (G) of spheres or colonies. Formation of colonies derived from G2 spheres in the presence of the AR antagonist Nilutamide is shown. (C) Western blot using Ab ERG 2805 of ERG protein within extracts of transgenic and WT G1 spheres. (D) Spheres formed from primary dissociated prostate cells were plated and grown in media containing the indicated drugs. Bicalutamide (Bic). Error bars correspond to ± s.d. (E) Sphere forming assays of KRT18^+^ B6WT and TMPRSS2-ERG^+^ cell lines with and without shRNA mediated ERG depletion. The number of spheres formed was counted at day 11. Invasion to FCS of the same B6 WT and TMPRSS2-ERG+ cell lines. Invasive cells in 5 microscopic fields (200×) were counted. All results shown are representative of at least 3 independent experiments. Error bars represent ±sem. ***P<0.001, **P<0.01, *P<0.05. Corresponding Western blot of ERG expression in the cell lines, using Ab ERG 2805, is also shown.

The majority of normal mouse prostate progenitor activity fractionates with cells expressing high Sca-1 [29], [30]. Similarly, sphere-forming activity was assayed in FACS separated transgenic prostate cell fractions, and virtually all of the sphere-forming units (SFU) were found in the lineage negative, Sca-1^+^/EpCAM^+^ fraction (Figure 5A). To determine whether TMPRSS2-ERG expression affects the self-renewal properties of prostate stem/progenitor cells, WT and fusion cell suspensions prepared from prostate tissue were assayed in parallel for sphere-forming units (SFU). There were about two fold more SFU per 10,000 nucleated primary generation 1 (G1) cells in the fusion as compared to the WT prostate, showing the existence of an increased pool of self renewing clonogenic cells in transgenic prostates (Figure 5B). There were no differences in the range of sizes or morphologies for wild type as compared to transgenic spheres. The average fold increase in G1 SFU for A5-derived cells was 2.4 (range 1.4–5.9, n = 4 independent experiments) and for H7-derived cells was 2.8 (range 1.3–6.5, n = 4 independent experiments). To measure intrinsic self-renewal activity, serial SFU activity was determined (Figure 5B). Fusion spheres continued to demonstrate about 2–3 times greater self-renewal activity compared to WT over 2 additional generations (G2 and G3). In addition, at each sphere generation, dissociated cells were assayed for colony forming units (CFU) as an independent measure of progenitor activity. Consistent with an intrinsic property, ERG protein was observed in transgenic protospheres (Figure 5C).

To evaluate signaling pathways that contribute to progenitor growth and survival, WT, *TMPRSS2-ERG*, *Pten^+/-^,* and *TMPRSS2-ERG*/*Pten^+/-^* prostates were evaluated for SFU activity and for responsiveness to drugs that inhibit select signaling pathways. *Pten^+/-^* prostates demonstrated increased SFU activity relative to WT, and additive SFU were observed in *TMPRSS2-ERG*/*Pten^+/-^* prostates, showing additional expansion of clonogenic cells in compound mutant mice (Figure 5D). Protospheres were incubated from their initiation with the AR antagonists, bicalutamide and nilutamide, or the AKT inhibitor, triciribine. Drug concentrations were titrated, and those shown represent the minimum concentration that leads to inhibition of WT sphere formation. The development of *TMPRSS2-ERG* and *Pten^+/-^* protospheres was decreased in the presence of AR antagonists or AKT inhibitors, while WT progenitor numbers were unchanged. These data suggest that early mutations such as *Pten* loss and *TMPRSS2-ERG* translocation lead to the presence of a unique self-renewing cell population. The drug sensitive phenotype was expressed in the EpCAM^+^/Sca-1^hi^ fraction (Figure 5A) and in serially-passaged SFU, showing that the presence of the drug-sensitive population appears to be an autonomous and intrinsic property of the fusion SFU (Figure 5B). Treatment with AR antagonists did not inhibit ERG expression in SFUs (Figure S2), suggesting that loss of ERG expression cannot account for the inhibition by nilutamide or bicalutamide and that AR functions in a parallel pathway to TMPRSS2-ERG in self-renewing progenitors. The intrinsic role of ERG in self-renewal was confirmed in KRT18^+^ cell lines established from WT and transgenic TMPRSS2-ERG primary prostate organoids, where presence of the fusion resulted in increased sphere forming activity as compared to wild type derived cells (Figure 5E). Introduction of independent shRNAs targeting ERG resulted in decreased sphere forming activity in transgenic cells, confirming ERG dependence. Previous studies have attributed increased invasion to upregulation of ERG expression [14], [15]. Similarly, in cell lines established from WT and transgenic TMPRSS2-ERG primary prostate organoids, TMPRSS2-ERG increased invasion compared to WT, and depletion of ERG resulted in decreased invasion of transgenic cells (Figure 5E). This data supports the concept that ERG is active within progenitor cells, and may elicit its effect through multiple mechanisms including increased self-renewal and/or dissemination.

### The TMPRSS2-ERG Promoter is Androgen-regulated in a Subpopulation of Prostate Epithelium

**Figure 6:**
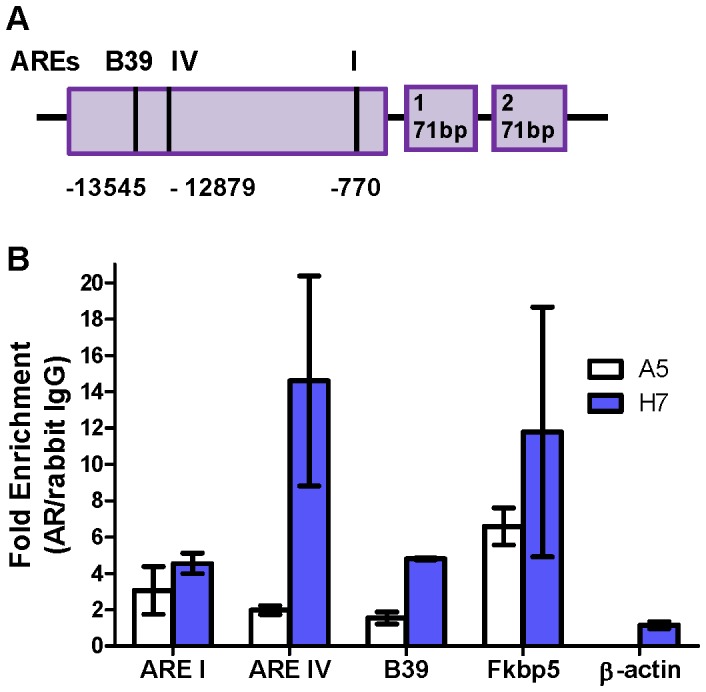
The introduced TMPRSS2 promoter binds AR in primary epithelial cells. (A) Schematic of the introduced human TMPRSS2 genomic region present within the BAC. The location relative to exon 1 (+1) of androgen response elements (ARE’s) that were bound by AR as presented in (B) are shown. (B) ChIP analysis for bound AR of fusion prostatic tissue (n = 2 for each line) *Fkbp5* and *ActB* are positive and negative controls, respectively, for AR binding. Fold enrichment corresponds to the QPCR signal in AR antibody samples relative to rabbit IgG controls. Error bars correspond to ± s.d.

**Figure 7:**
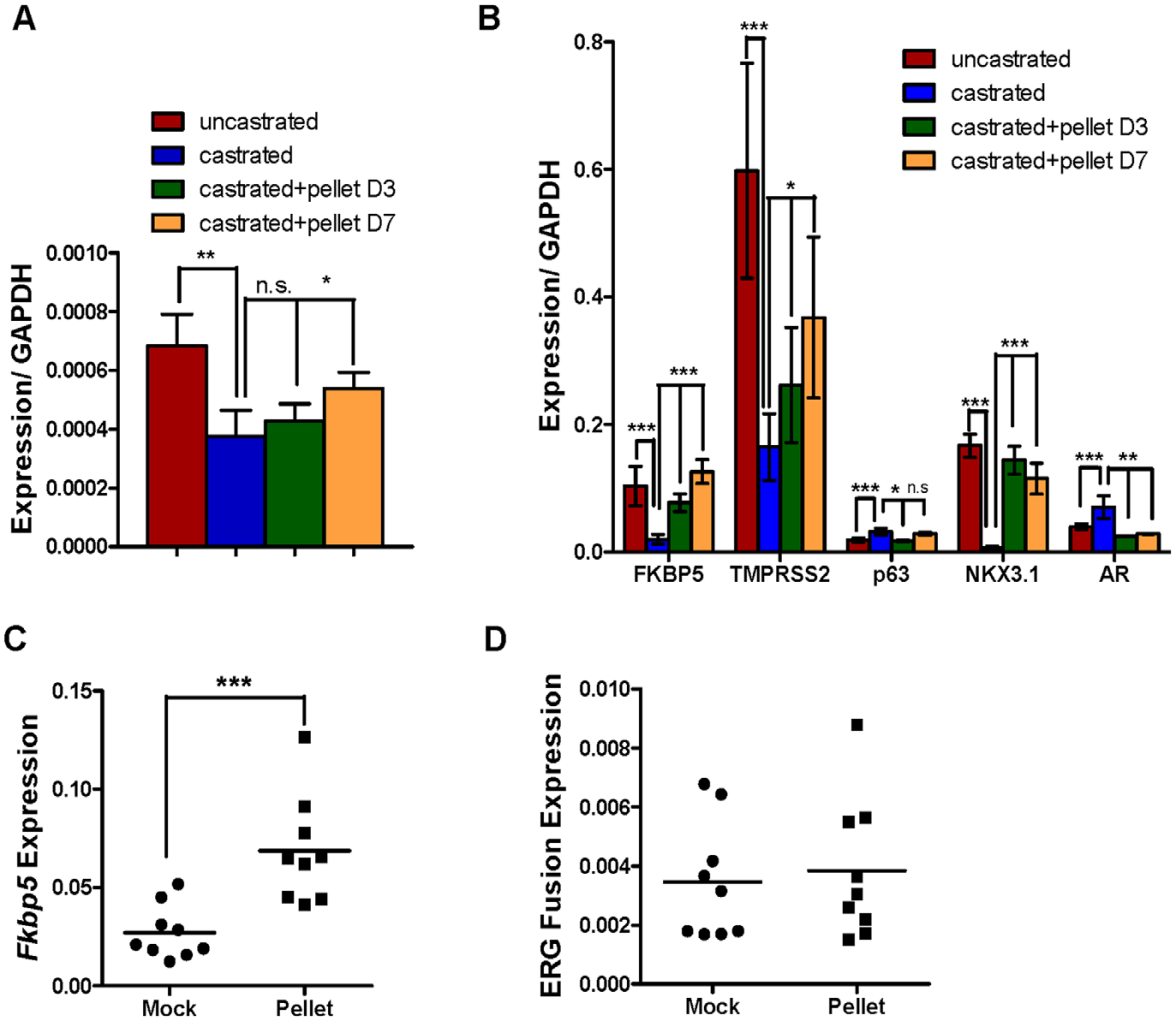
*TMPRSS2* is expressed in castration-sensitive and resistant populations and is not responsive to supraphysiological concentrations of androgen. (A) QRT-PCR expression analysis of ERG fusion transcripts using primer pairs b/d (Figure 1A) in A5 transgenic prostates isolated from uncastrated (n = 8), castrated (n = 9), and castrated animals with subsequent androgen supplementation, where tissue was harvested at days 3 (n = 3) or 7 (n = 3) post pellet implantation. (B) QRT-PCR expression analysis of lineage markers and known AR regulated genes from the samples described in (A). Error bars correspond to ± s.d, (C-D) QRT-PCR analysis of RNA isolated from prostate tissue with (squares) or without (circles) androgen pellet supplementation for 3 days was performed, and the levels of expression following normalization to *Gapdh* are shown. Symbols represent individual animals. Expression of (C) *Fkbp5* and (D) the TMPRSS2-ERG fusion, assessed using primers b/d (Figure 1A), are shown. ***P<0.001, **P<0.01, *P<0.05.

We investigated the regulation of the TMPRSS2-ERG fusion by androgen in primary prostate epithelial cells. Using ChIP analysis performed on whole prostates, we confirmed that androgen receptor (AR) bound to previously described sites (AREI, AREIV, and B39) in the transgenic human *TMPRSS2* promoter (Figure 6) [21], [22]. These data suggest that appropriate chromatin organization leading to AR association was established in the TMPRSS2-ERG BAC transgenic prostates. To assay the regulation by androgen of *TMPRSS2-*driven *ERG,* the expression of fusion RNA was quantified in prostates following castration (day 14) and subsequent androgen supplementation for 3 or 7 days. As shown in Figure 7A, the *ERG* fusion transcript level was reduced by about half and subsequently rebounded upon androgen supplementation. This pattern is similar to that of endogenous *Tmprss2* but contrasts with the nearly complete loss of RNA encoding *Fkbp5* and *Nkx3.1* (Figure 7B). These data suggest that both castration sensitive and resistant populations express *TMPRSS2-ERG* and *Tmprss2*.

In addition, *TMPRSS2-ERG* and *Fkbp5* RNA levels were assayed in whole prostate in response to a supraphysiological concentration of androgen for 3 days. Consistent with the castration studies, *Fkbp5* demonstrated androgen induction. However, the *ERG* fusion-specific transcript was not increased (Figures 7C&D).

## Discussion

Here we describe a BAC transgenic model of TMPRSS2-ERG translocation, which uniquely has addressed *TMPRSS2*-driven *ERG* transcriptional regulation and phenotype in primary prostate tissue and early lesions. Consistent with previous studies using Pb-driven *ERG,* TMPRSS2-ERG alone does not appear to lead to any obvious pathological changes, but accelerates the initiation of prostatic neoplasia in combination with heterozygous *Pten* loss [15], [16]. Our results demonstrating that TMPRSS2-ERG accelerates PIN development but not progression to adenocarcinoma are in agreement with the Pb-driven *ERG* cDNA model of King et al. [16]. Interestingly, in comparison to the VCaP cell line, we observed similar levels of *ERG* RNA but decreased levels of steady-state ERG protein. These data suggest that a post-transcriptional mechanism regulating ERG protein level exists. One such mechanism that decreases protein degradation of the Ets family member, ETV1, has been described in gastrointestinal stromal tumors [33], [34]. It is reasonable to speculate that mutations contributing to prostate cancer progression may influence ERG protein stability.

Transgenic TMPRSS2-ERG was expressed in most luminal and a fraction of basal epithelial cells as determined by FACS fractionation and tissue staining. Thus, the extended human TMPRSS2 promoter can be expressed in multiple prostate epithelial cell types. The expression of TMPRSS2-ERG in the EpCAM^+^/Sca-1^hi^ fraction, which includes basal and stem/progenitor cells, was associated with a relative increase in clonogenic cells. Two potential mechanisms that could contribute to the expansion of SFU are increased progenitor production and/or impaired differentiation, resulting in the accumulation of a partially-differentiated transit-amplifying population. Consistent with this concept, the transgenic compared to the wild type progenitors expressed a unique phenotype, increased sensitivity toward inhibition of either AR or AKT signaling pathways. Interestingly, the KRT18^+^ ERG^+^ cell line demonstrated ERG-dependent clonogenic and invasive activity, supporting the concept that ERG contributes to the amplification of preneoplastic prostate epithelial cells.

A comprehensive understanding of *TMPRSS2* transcriptional regulation is important in considering the effect of androgen deprivation therapy upon prostate cancers harboring *TMPRSS2-ERG* fusions. Although *TMPRSS2* is expressed at the highest relative levels in prostate epithelium, *TMPRSS2* is robustly expressed in other tissues as well, suggesting that AR is not necessary for its transcription [35], [36]. The regulation of *TMPRSS2* by AR has been shown directly in a limited number of AR+ luminal prostate cancer cell lines and indirectly in primary prostate cancers where *TMPRSS2* expression correlates with expression of other AR-regulated genes [21], [22], [37], [38]. In this study, castration and subsequent androgen supplementation, demonstrated *TMPRSS2-*driven ERG fusion was partially androgen-dependent, significantly less so than *Fkbp5* and *Nkx3.1*. We conclude that in primary prostate epithelial cells, some TMPRSS2 expression occurs under castrate conditions. Analysis of *TMPRSS2-ERG* positive CRPC clinical samples has revealed examples of *ERG* expression which were relatively more robust than other AR-regulated genes such as *Nkx3.*1 [39].

In conclusion, the studies presented here provide novel data concerning the role and regulation of *TMPRSS2-ERG* in vivo. Additional studies using TMPRSS2-ERG models will aid in defining basic mechanisms of action and of synergism with defined mutations, as well as helping to establish accurate preclinical models for prostate cancer treatment.

## Materials and Methods

### Ethics Statement

All animal experiments were done according to the protocol (LCB-018) approved by the National Cancer Institute Guideline for Use and Care of Animals.

### TMPRSS2-ERG BAC Transgenesis

For recombineering, prior to the two recombination steps, a base pBR322 vector was constructed by traditional cloning which contained a neomycin selection cassette and TMPRSS2 and ERG homology segments ranging from 415–470 bp in size. The 229.466 Kb TMPRSS2-ERG BAC was generated using the mini-ë Red Recombination system [25]. Briefly, the mini-ë DNA was electroporated into DH10B cells containing the TMPRSS2 BAC, RP11-709L15 (BACPAC Resources Center, Children’s Hospital Oakland Research Institute) and selected on LB plates containing tetracycline. Expression of the phage genes necessary for recombination, exo, bet and gam, was induced at 42°C and linearised pBR322 base vector was electroporated into the cells containing the TMPRSS2 BAC and the mini-ë DNA. Positive recombination events, where the required region of TMPRSS2 BAC was inserted into the pBR322 vector, were selected on LB plates containing Kanamycin (50 ug/ml). The pBR322/TMPRSS2 construct was confirmed by restriction digest analysis. Linearised pBR322/TMPRSS2 DNA was then electroporated into induced DH10B cells containing the ERG BAC (CTD-2511E13) and mini-ë DNA. Positive recombination, where the TMPRSS2 region was inserted prior to 45016 bp of the ERG BAC resulting in a 229.466 Kb construct, was confirmed by pulse field electrophoresis. Transgenic animals were generated on the FVB and C57/BL6 backgrounds where positive founders were identified by southern blot using a radiolabelled probe generated from the pBR322/TMPRSS2 construct (Laboratory Animal Sciences Program, NCI-Frederick). Germline transmission was determined using primers b/c (Figure 1A).

#### Other Strains

The *Pten* ^+/-^ strain on a C57/BL6 background and the *Nkx3.1^+/-^* strain on the FVB background were both obtained from MMHCC (Frederick, MD).

### Primary Prostate Cell Preparation

Prostate lobes were dissected from the urogenital tracts of male mice. Following mechanical dissociation, the tissue was digested with rotation for 2 h at 37°C in DMEM with 10% fetal bovine serum (FBS), 0.2 mg/ml DNase I (Sigma), and 1 mg/ml of collagenase D (Roche) or 270 U ml^−1^ collagenase II (Sigma) for organoid or single cell dissociation respectively. Organoids were washed once with PBS, resuspended in WIT-P medium (Stemgent) and further dissociated by passing through a 19-gauge needle. For single cell dissociation, the culture was resuspended in 0.05% trypsin-EDTA for 3–5 min and subsequently in prostate epithelial cell basal media (PrEGM) containing bovine pituitary extract, insulin, hydrocortisone, gentamicin, amphotericin B, retinoic acid, transferrin, triiodothyronine, epinephrine and recombinant human epidermal growth factor. A single cell suspension was obtained by passing the suspension serially through 19–30.5 gauge needles and finally through a 40-µM strainer. Organoids were plated into 6-well Primaria plates (BD Bioscience) in WIT-P medium. Medium was changed after three days when a monolayer of prostate epithelial cells was established.

### Derivation of Cell Lines

Prostate lobes were isolated from C57/BL6 wild-type (B6 WT) or C57/BL6 TMPRSS2-ERG transgenic male mice. Organoids were obtained as described above and cultured in WIT-P medium (Stemgent, Cambridge, MA). Monolayers of prostate epithelial cells were trypsinized after 7 days, and passed through continuous generations until cell lines were established. FACS analysis established that >90% of cells were KRT18^+^/KRT5^−^.

### In vitro Transient Expression

Nearly full length TMPRSS2-ERG cDNA was amplified using primers a/h (Figure 1B) and then inserted in frame into the pcDNA4/V5-His-A vector using standard cloning techniques. Resulting clones were sequenced and expressed transiently in Cos 7 cells using Lipofectamine Plus reagents (Invitrogen).

### shRNA Lentiviral Infection

HEK293FT cells were infected with the following DNA: 2.0 ug of VSV-G, 6.0 ug of PsPAXII and 8.0 ug of either pGipZ empty vector RHS4349, ERG shRNA 1- V3LHS-387037 or ERG shRNA 2- V3LHS-412525 (Open Biosystems). The derived transgenic TMPRSS2-ERG cell line was infected using the viral supernatant, and selected with puromycin (0.5 ug/ml).

### In vitro Invasion

Invasion chambers were prepared as described previously [40] except that growth factor–reduced Matrigel (BD Bioscience) was used and cell invasion was carried out as outlined [41].

### Statistical Analysis

Analysis was performed within GraphPad Prism, where unpaired t-test was performed and the SEM or SD was calculated. The p values obtained are indicated.

### Chromatin Immunoprecipitation (ChIP)

ChIP assay was performed using the EZ magna ChIP A kit (Millipore) with a modified protocol. Prostates were harvested, dissected from the urogenital tract, and snap frozen in liquid nitrogen, followed by pulverizing and cross-linking with 1% formaldehyde at RT for 15 min. The fixation was quenched with glycine, and cells were washed twice with cold PBS containing Complete Protease Inhibitor (Roche). Cell pellets were resuspended in cell lysis buffer and incubated on ice for 15 min. Nuclei were collected by centrifugation at 10,000 rpm at 4°C for 10 min. Nuclei were then resuspended in nuclei lysis buffer and chromatin was sheared using a bath sonicator (Diagenode Bioruptor) at high power for 40 min with on and off cycling alternating every 30 sec. Sheared chromatin was divided to perform immunoprecipitation with rabbit IgG antibody (Millipore) or anti-AR antibody (N-20; Santa Cruz) on each individual prostate sample. Immunoprecipitation, washing, elution, reverse cross-linking, and DNA purification steps were performed according to manufacturers’ instructions (Millipore). Quantitative PCR was performed with 2 µl of eluted chromatin in triplicate. ChIP PCR primers for the human *TMPRSS2* promoter and enhancer region and for the *Fkbp5* enhancer region were adopted from published data [22], [42] and are listed in Table S1. *ActB* served as a negative control.

### Castration and Androgen Pellet Implantation

Mice were castrated through an abdominal incision, and following 14 days were implanted subcutaneously with a 12.5 mg testosterone pellet (Innovative Research of America). Prostates were harvested at day 0, day 3 and day 7 post pellet implantation, dissected from the urogenital tract, and snap frozen in liquid nitrogen. For investigation of androgen response in intact mice, animals were surgically implanted with a 12.5 mg testosterone pellet on the back or underwent mock surgery. Three days later, prostates were harvested. Total RNA extraction and quantitative RT-PCR was performed as described below. *Fkbp5*, a known AR responsive gene, was considered as a positive control gene. Primers are listed in Table S1.

### Reverse Transcription-PCR

Total RNA was isolated, according to manufacturers’ instructions, from prostate tissue using Trizol (Invitrogen), from organoids using the RNeasy Mini Kit (Qiagen), and from in vitro and FACS sorted cells using the RNeasy Micro Kit (Qiagen). cDNA was generated from total RNA derived from prostate tissue and organoid cultures utilizing the Super Script III First Strand Synthesis System for RT-PCR (Invitrogen). For in vitro and FACS sorted cells, the WT-Ovation RNA amplification system was used to generate cDNA (Nugen). PCR was performed using platinum taq polymerase (Invitrogen). SYBR Green Mastermix or Taqman Gene Expression Master Mix was used for quantitative RT-PCR on the Stepone Plus RT PCR System (Applied Biosystems). All reactions were run in triplicate using primers listed in Table S1. Primer 3 or Genscript software was used for primer design. Values were normalized to *Gapdh* unless otherwise stated.

### Western Blot Analysis

Cells were lysed with RIPA buffer containing Complete Protease inhibitors (Roche) and the phosphatase inhibitors, 25 mM â-glycerophosphate, 10 mM sodium fluoride and 1 mM sodium orthovanadate. Primary antibodies were incubated overnight at 4°C using the following dilutions, ERG 2805- 1∶2000 (Epitomics); mAb ERG CPDR- 1∶500 [10]; ERG 5115- 1∶2000 (Epitomics);V5- 1∶5000 (Invitrogen) and Tubulin- 1∶2500 (Sigma). Either horseradish peroxidase conjugated anti mouse IgG or anti rabbit IgG secondary antibodies were used and developed with Super Signal West Pico or Femto Chemiluminescent Substrate (Thermo Scientific).

### Immunofluorescence and Confocal Microscopy

Tissue was fixed in a 4% paraformaldehyde-PBS for 2 h incubated in 15% sucrose for 2 h and stored overnight in a 30% sucrose solution. Tissue was embedded in OTC, frozen in an ethanol dry ice bath, and sectioned at 7 µM. Immunofluorescence was carried out on the frozen tissue sections. The tissue sections were incubated in methanol containing 0.3% H_2_O_2_ for 30 min at 4°C. For ERG Antibody 2805 (Epitomics) staining alone, non-specific staining was blocked by incubation in 20% goat serum, 2% BSA in PBS (GSB) for 40 min at room temperature, followed by an overnight incubation in primary antibody at 4°C. Following washes, the sections were incubated with a fluorescently tagged secondary antibody (Invitrogen) for 30 min at room temperature. Antibodies were diluted in GSB. The sections were mounted using Fluoro-Gel with DAPI (EMS). For ERG Antibody 2805 and CK8 (Covance) co-staining, the ERG staining was performed as above. The CK8 staining was performed using the Mouse on Mouse Fluorescein Kit (Vector Laboratories) standard protocol following ERG staining. For ERG Antibody 2805 and TP63 (Millipore) co-staining, the tissue sections were subjected to steam antigen retrieval for 15 min using Target Retrieval solution (Dako North America). Non-specific staining in the tissue sections was blocked by incubating the sections with GSB for 40 min at room temperature, followed by an overnight incubation with both primary antibodies at 4°C. Following washes, the sections were incubated with fluorescently tagged secondary antibodies (Invitrogen) for 30 min at room temperature. Antibodies were diluted in GSB. The sections were mounted using Fluoro-Gel with Dapi (EMS). For all stains the antibody dilutions were as follows; ERG Antibody 2805, 1∶25; TP63, 1∶200; CK8, 1∶200. Stained tissues were visualized on a Zeiss AxioObserver.Z1 inverted microscope equipped with a 20× Pln Apo/0.8 DIC II objective. Confocal images of stained tissues were generated on a Zeiss LSM 510 META scanning laser microscope.

### Histological Assessment

The urogenital tracts of male mice were removed, fixed in 4% paraformaldehyde in PBS overnight, transferred to 70% ethanol, trimmed and embedded in paraffin, and 5 µm sections produced. Hematoxylin and eosin staining was performed (Histoserv Inc.). Foci of prostate hyperplasia were characterized by proliferation of hypertrophied epithelial cells with a columnar morphology and enlarged nuclei. Mouse prostatic intraepithelial neoplasia (mPIN) lesions were characterized by neoplastic proliferation of the epithelium that partially or completely occluded the glandular lumen, without invasion of the basement membrane. The histological pattern was cribiform or solid, with cytological and nuclear atypia (cytomegaly, anistocytosis, anisokaryosis, karyomegaly, abnormal chromatin pattern, and prominent nucleoli). Occasional mitoses and multinucleated cells were observed. Foci of invasive adenocarcinoma were characterized by individualized or nests of undifferentiated round to polygonal neoplastic cells and small glands extending into the smooth muscle and desmoplastic stroma beyond the basement membrane of mPIN affected glands. Mitotic figures averaged less than 1 per 10 HPF. Moderate numbers of small lymphocytes, plasma cells, histiocytes and fewer neutrophils occasionally admixed with necrotic debris were scattered throughout foci and surrounding stroma. Invasion was confirmed by examining serial sections.

### Cell labeling and FACS Sorting

For labeling reactions, cells were resuspended in PBS (without Mg^2+^ or Ca^2+^) containing 1% heat-inactivated FBS, and 0.09% (w/v) sodium azide. All antibodies and reagents were purchased from BD Pharmingen unless otherwise stated. Fcγ III/II receptors were blocked using anti-CD16/CD32 antibody for 15 min at 4°C. Cells were stained for lineage depletion with CD45-FITC, CD31-FITC, Ter-119-FITC, and for separation with Sca-1-PE (clone E13-161.7) and EpCAM-APC (clone G8.8) (Biolegend) antibodies for 30 min at 4°C. The 7-aminoactinomycin D (7-AAD) (Sigma) 100 µg ml^−1^ was added prior to analysis. Cell sorting was performed on FACSVantage and FACSAria cell sorters (Becton Dickinson) using FACSDiva software. The FACS gating strategy for Sca-1/EpCAM is shown in Figure S3. Post-sort purity of individual fractions was assessed and purities of >90% were routinely achieved.

**Colony and Sphere Forming Assays** were Performed as Previously Described [43].

## Supporting Information

Figure S1 **Investigation of potential cross reactivity of Ab 2805 in prostate epithelial cells.** To address possible cross reactivity of Ab 2805 with the Ets family member, *Fli-1* in epithelial cells of the prostate, QRT-PCR analysis of *Fli-1* expression in FACS sorted Sca-1/EpCAM fractions was performed. The stromal fraction expressed *Fli-1.* The luminal EpCAM^+^ fraction was negative for *Fli-1* and a minimal signal was observed in Sca-1^hi^/EpCAM^+^, which is likely due to a minor number of EpCAM^−^ positive cells in the fraction (Figure 3A).
(TIF)

Figure S2 **ERG expression is not altered in spheres treated with AR antagonists or an Akt inhibitor.** RNA was isolated at day 14 from H7 spheres cultured in media alone or media amended with the AR antagonists, Nilutamide (Nil) and Bicalutamide (Bic) or the Akt inhibitor Triciribine (Tricir). QRT-PCR was performed on amplified samples using ERG fusion primers, b/d (Figure 1A).
(TIF)

Figure S3 **FACS gating strategy used to isolate Sca-1/EpCAM double positive cells from transgenic and wild type murine prostate tissue.** FACS plots show (A) forward/side scatter and the gates used to identify (B) viable (blue), (C) lineage negative (green), (D) Sca-1/EpCAM double positive (red) cells.
(TIF)

Table S1 **Primer sequences utilized in QRT-PCR and CHIP Q-PCR reactions are shown.** In addition to the use of previously described PCR primer sequences, the software programs Primer 3 and Genscript were used to design PCR primers.
(DOC)
