# Supplementary figures and images for: TMPRSS2- Driven ERG Expression In Vivo Increases Self-Renewal and Maintains Expression in a Castration Resistant Subpopulation
Source: PLoS One. 2012 Jul 30;7(7):e41668. doi: 10.1371/journal.pone.0041668 (PMC3408501; doi:10.1371/journal.pone.0041668)

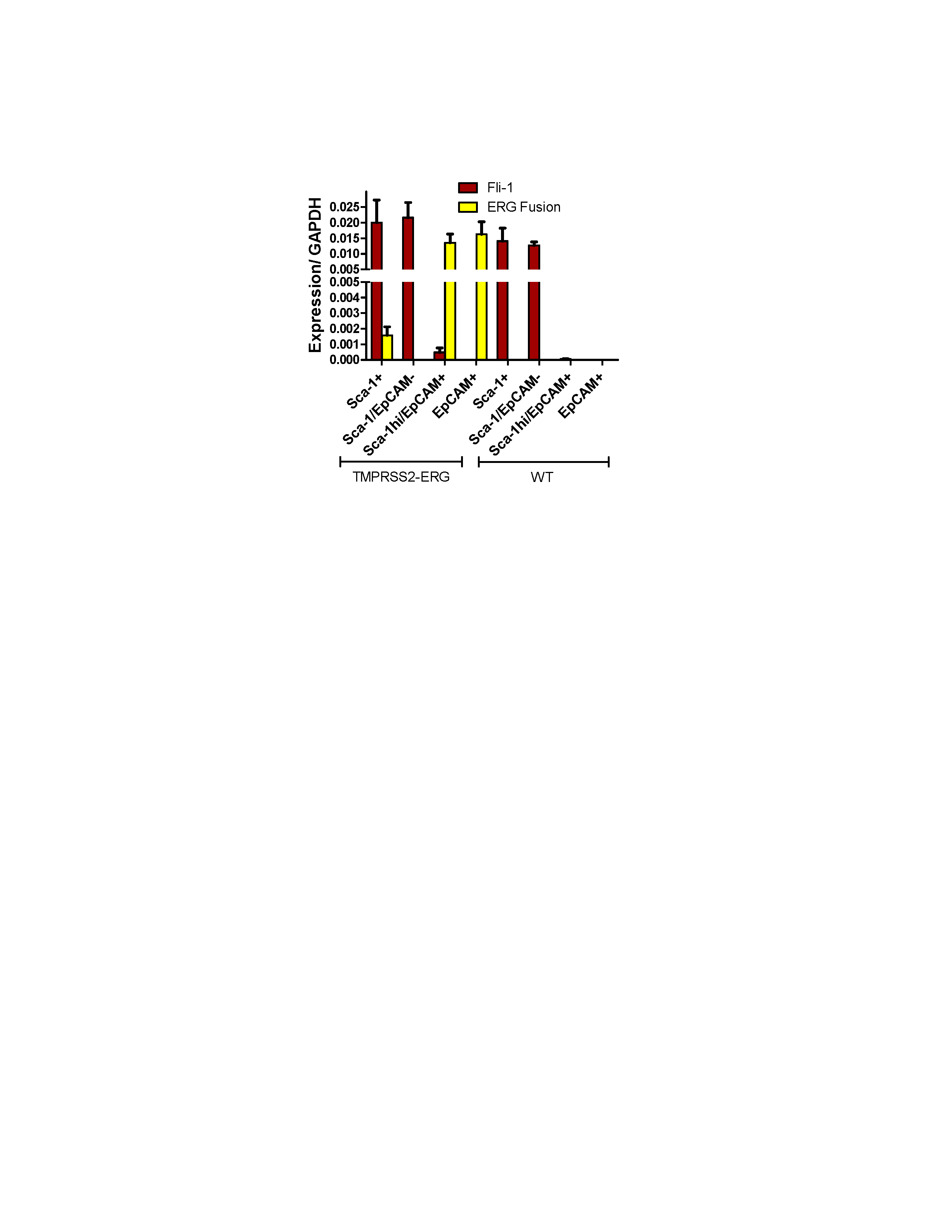

Supplement: Figure S1 — Investigation of potential cross reactivity of Ab 2805 in prostate epithelial cells. To address possible cross reactivity of Ab 2805 with the Ets family member, Fli-1 in epithelial cells of the prostate, QRT-PCR analysis of Fli-1 expression in FACS sorted Sca-1/EpCAM fractions was performed. The stromal fraction expressed Fli-1. The luminal EpCAM+ fraction was negative for Fli-1 and a minimal signal was observed in Sca-1hi/EpCAM+, which is likely due to a minor number of EpCAM− positive cells in the fraction (Figure 3A). (TIF) [file pone.0041668.s001.tif]

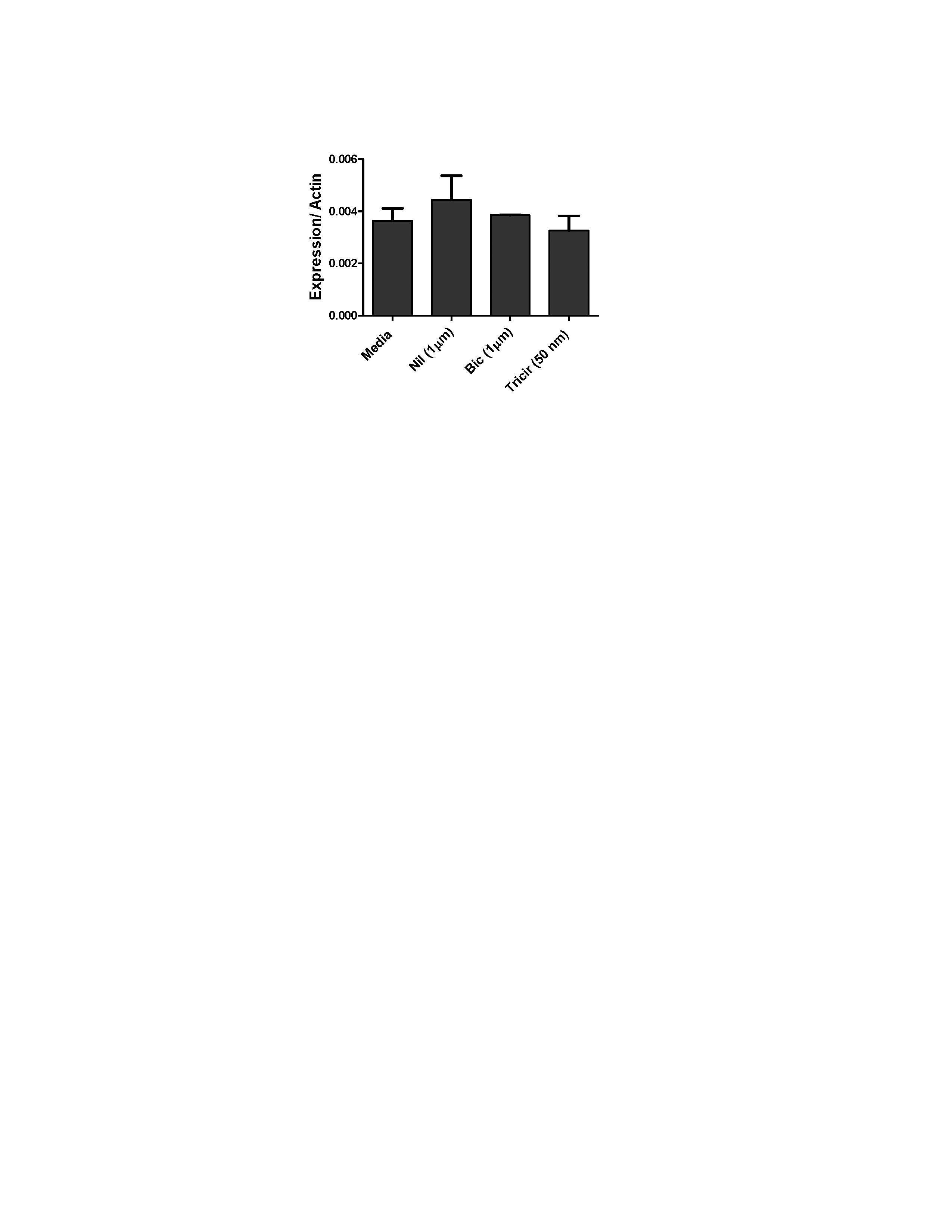

Supplement: Figure S2 — ERG expression is not altered in spheres treated with AR antagonists or an Akt inhibitor. RNA was isolated at day 14 from H7 spheres cultured in media alone or media amended with the AR antagonists, Nilutamide (Nil) and Bicalutamide (Bic) or the Akt inhibitor Triciribine (Tricir). QRT-PCR was performed on amplified samples using ERG fusion primers, b/d (Figure 1A). (TIF) [file pone.0041668.s002.tif]

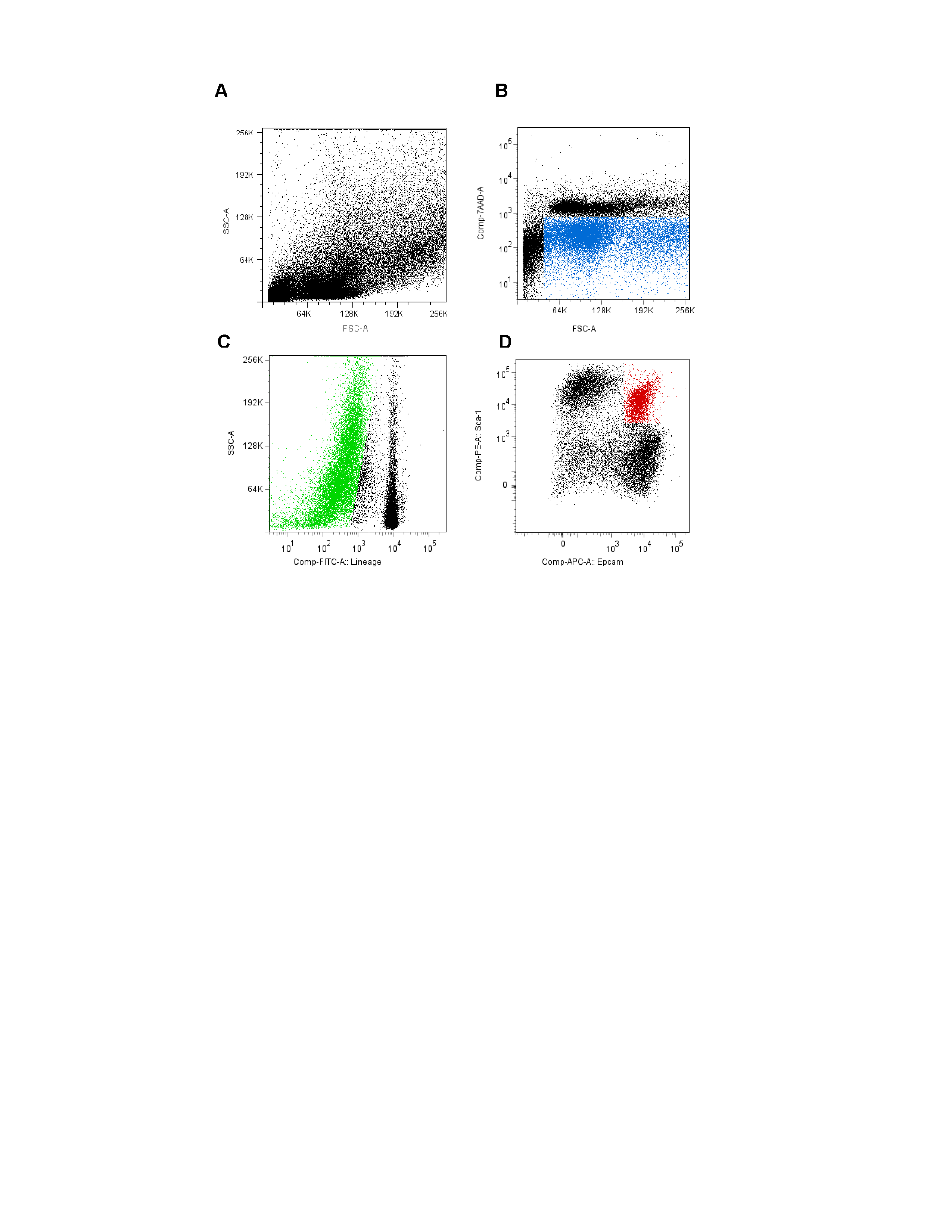

Supplement: Figure S3 — FACS gating strategy used to isolate Sca-1/EpCAM double positive cells from transgenic and wild type murine prostate tissue. FACS plots show (A) forward/side scatter and the gates used to identify (B) viable (blue), (C) lineage negative (green), (D) Sca-1/EpCAM double positive (red) cells. (TIF) [file pone.0041668.s003.tif]
