# Supplementary material for: TMPRSS2- Driven ERG Expression In Vivo Increases Self-Renewal and Maintains Expression in a Castration Resistant Subpopulation
Source: PLoS One. 2012 Jul 30;7(7):e41668. doi: 10.1371/journal.pone.0041668 (PMC3408501; doi:10.1371/journal.pone.0041668)
Supplement: Table S1 — Primer sequences utilized in QRT-PCR and CHIP Q-PCR reactions are shown. In addition to the use of previously described PCR primer sequences, the software programs Primer 3 and Genscript were used to design PCR primers. (DOC) [file pone.0041668.s004.doc]

**Table S1**. Primers

| **QRT-PCR primers** | **5'-3'** | **Key Figure 1A** |
| --- | --- | --- |
| Gapdh For | CAGAACATCATCCCTGCATC |  |
| Gapdh Rev | CTGCTTCACCACCTTCTTGA |  |
| Fkbp5 For | CTGTGGTGGAAGGACATTTG |  |
| Fkbp5 Rev | AAACCATAGCGTGGTCCAA |  |
| Nkx3.1 For | GGAGAGGAAGTTCAGCCATC |  |
| Nkx3.1 Rev | TGGCAAAGACAATGGTGAGT |  |
| Tmprss2 For | GCTGTCTTGCTTTGGAGGTT |  |
| Tmprss2 Rev | CCGTCACACCAGAGAGAAGA |  |
| p63 For | CAGTCAAGCACTGCCAAGTC |  |
| p63 Rev | CATCACCTTGATCTGGATGG |  |
| mAR For | GTTGGCGGTCCTTCACTAAT |  |
| mAR Rev | CTCATCCTCACACACTGGCT |  |
| Fli-1 For | GCCAACCCCATGAACTAT |  |
| Fli-1 Rev | GCAGGCACAATGACTCTC |  |
| Vimentin For | GATGCGTGAGATGGAAGAGA |  |
| Vimentin Rev | GGCCATCTTAACATTGAGCA |  |
| TMPRSS2 Exon 1 For | CTAAGCAGGAGGCGGAGGCGGAG | a |
| TMPRSS2 Exon 2 For | GATAACAGCAAGATGGCTTTGAACTCA | b |
| ERG Exon 8 Rev | TCAAACAACGACTGGTCCTCACTCAC | c |
| ERG Exon 9 Rev | TGCCGTAGTTCATCCCAACG | d |
| ERG Exon 11 For | TTCAGATGATGTTGATAAAGCCTTACA | e |
| ERG Exon 13 Rev | TCCAGGCTGATCTCCTGGG | f |
| ERG Exon 16 For | CCCACCCACAGAAGATGAAC | g |
| ERG Exon 16 Rev | ATTCCAGTATGGGTTTGGGGCAGC | h |
| ERG Exon 12 For | AGGGGGTGCAGCTTTTATTT | i |
| ERG8 Rev  ERG FAM | GACTTAGGGCACGGATCTCA  ATGCATGCTAGAAACACAGATTTACCA | J |
| **CHIP Q-PCR primers** | **5'-3'** |  |
| mFkbp5 ARE 6/7 For | ACCCCCATTTTAATCGGAGAAC |  |
| mFkbp5 ARE 6/7 Rev | TTTTGAAGAGCACAGAACACCCT |  |
| hTMPRSS2 ARE I For | CTGAGCCCCCACAATTGC |  |
| hTMPRSS2 ARE I Rev | GGTGGGACACACCTCAGCC |  |
| hTMPRSS2 ARE IV For | TCCCAAATCCTGACCCCA |  |
| hTMPRSS2 ARE IV Rev | ACCACACAGCCCCTAGGAGA |  |
| hTMPRSS2 B39 For | TCCAGGCAGAGGTGTGGC |  |
| hTMPRSS2 B39 Rev | CGTATGTCTCCCTGCACCACT |  |
| Beta-actin For | TCCTCCCTGGAGAAGAGCTA |  |
| Beta-actin Rev | ACGGATGTCAACGTCACACT |  |
